# Stable Ruptured Thoracic Aortic Aneurysm

**DOI:** 10.7759/cureus.93235

**Published:** 2025-09-25

**Authors:** Ahmed Mahmood, Samia Sarwar, Ayman Mahmoud Hamouda

**Affiliations:** 1 Accident and Emergency, National Hospital and Medical Center, Lahore, PAK; 2 Accident and Emergency, Pilgrim Hospital, Boston, GBR; 3 Community Health Sciences, Fatima Memorial Hospital College of Medicine and Dentistry, Lahore, PAK

**Keywords:** artery aneurysm rupture, descending aorta aneurysm, rupture of aneurysm, thoracic aorta, thoracic aortic aneurysm (taa)

## Abstract

A ruptured aortic aneurysm is a life-threatening condition that should be dealt with promptly and requires early management by a concerned specialist team. A ruptured aortic aneurysm can present with a wide array of symptoms, ranging from shortness of breath, chest pain, to sudden collapse. This is a case of a female in her 70s who presented to the emergency department after an unwitnessed collapse at home. Following early tests, it was determined that the patient had a ruptured thoracic aortic aneurysm (TAA), which was deemed to be stable. She was then referred to the vascular team for additional treatment.

## Introduction

Acute aortic syndrome (AAS) is a life-threatening condition involving the largest vessel of the body, the aorta. This syndrome encompasses various conditions, including a thoracic aortic aneurysm (TAA), aortic dissection, intramural haematoma, and penetrating aortic ulcer. A TAA is defined as a swelling or bulge in the weakest section of the main blood vessel coming from the heart, known as the aorta. Hence, it is susceptible to expansion, tearing, dissection within the wall, and ultimately rupture.

It is mostly asymptomatic, but when symptoms do appear, they typically include chest pain, shortness of breath, back pain, low blood pressure, and even loss of consciousness [1]. In some instances, a TAA can present atypically, causing symptoms of dysphagia, hoarseness of voice, or even features of heart failure such as cough, shortness of breath, and leg swelling due to compression of surrounding structures. These unusual presentations can often result in a delay in management. Male gender, smoking, atherosclerosis, high blood pressure, high cholesterol levels, predisposing genetic disorders, blood vessel inflammation, and untreated infections, in some instances as a congenital disorder itself and, in rare cases, trauma, can all contribute to TAA formation [1,2].

TAAs can cause fatal complications, such as aortic dissection or rupture, and are thus known as AAS and aortic arch syndrome or “silent killers” [3,4,5]. Up to 22% of people who suffer an aortic arch complication die at home before receiving medical assistance, and 34% die within the first 30 days after being at the hospital [4]. Hence, early diagnosis, including atypical presentations such as this case, is critical for effective management and a better prognosis.

## Case presentation

A lady in her 70s was brought to the emergency department by ambulance after being discovered lying on the floor by her morning caregiver around 10:00 (10:00 am). She was last seen on the bed by a nighttime caregiver at 21:00 (9:00 pm) the previous day. She was unable to recall events that may have led her to be on the floor and was deemed to lack capacity with the potential diagnosis of acute delirium. Initial differential diagnoses of acute delirium included infections of the lower respiratory tract or urinary tract, intracranial haemorrhage, acute coronary syndrome, orthostatic hypotension, and rhabdomyolysis.

During the initial triage at 11:00 (11:00 am), she had a national early warning system (NEWS) score of 1, a heart rate of 95 beats per minute, a respiratory rate of 19 breaths per minute, a temperature of 36.7 degrees celsius, a blood pressure of 137/94 mm of mercury, and an oxygen saturation of 97% at room air. Her physical examination of the respiratory system revealed bilateral coarse crepitus on auscultation. However, the remainder of the examinations, including neurological, cardiac, and abdominal examinations, were found to be unremarkable. There was no substantial postural drop in blood pressure, and it remained within normal limits in both arms. Her past medical history included advanced Alzheimer’s dementia, hypertension, Crohn’s disease, and gastroesophageal reflux disease. Her personal history included being a smoker and living alone with carers visiting four times per day.

**Figure 1:**
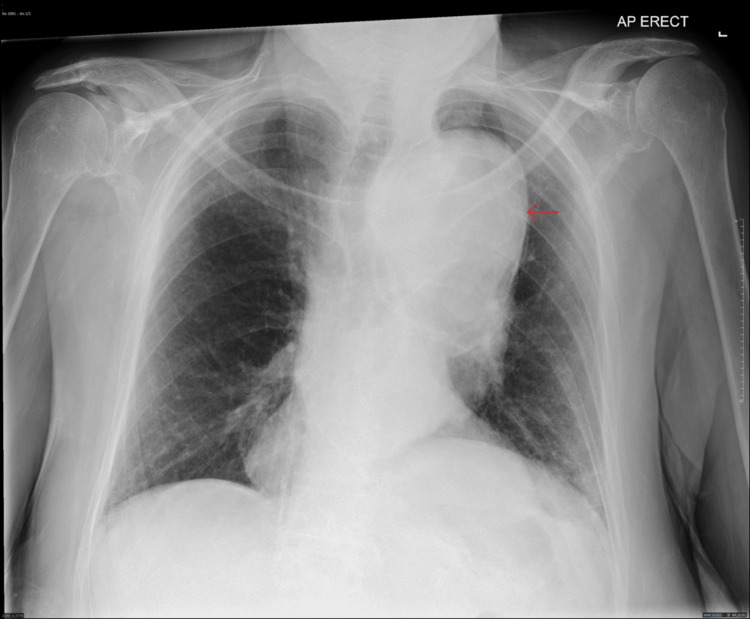
Chest X-ray A large thoracic aortic aneurysm was found in the region of the aortic arch, which was not evident on previous examination as highlighted by the red arrow.

**Figure 2:**
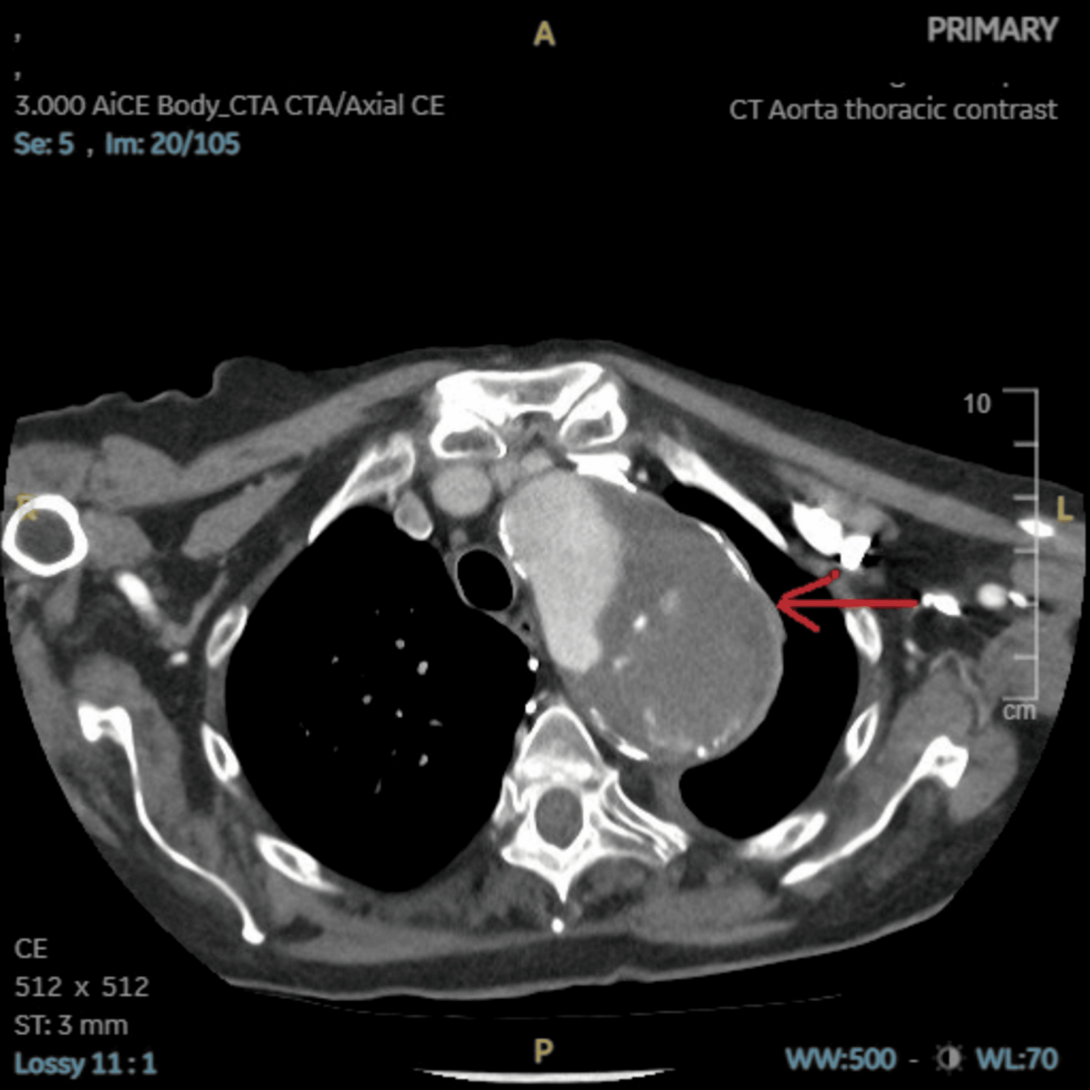
CT aorta with contrast axial view showing a large ruptured aneurysm as highlighted by the red arrow.

**Figure 3:**
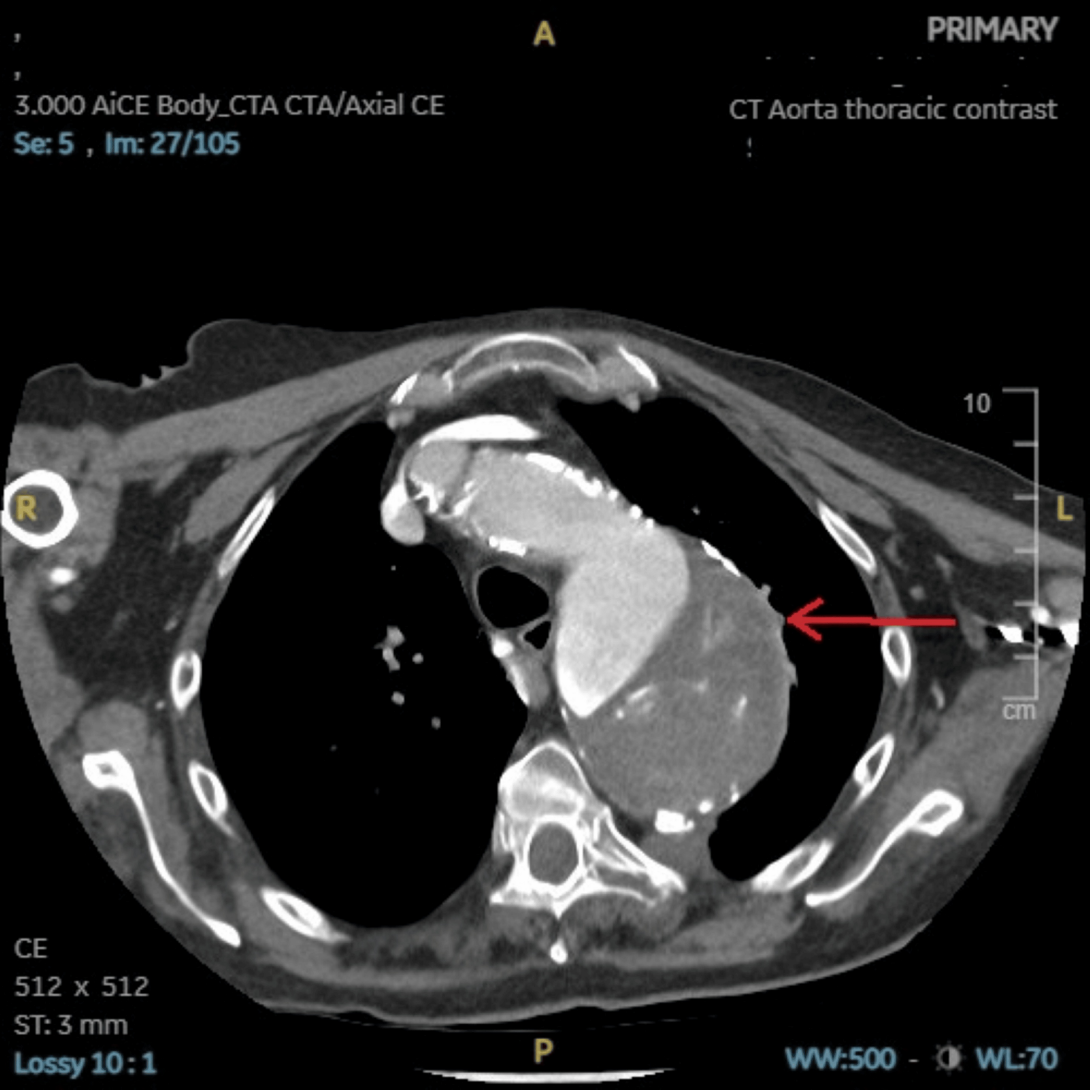
CT aorta with contrast axial view showing a large ruptured aneurysm as highlighted by the red arrow.

**Figure 4:**
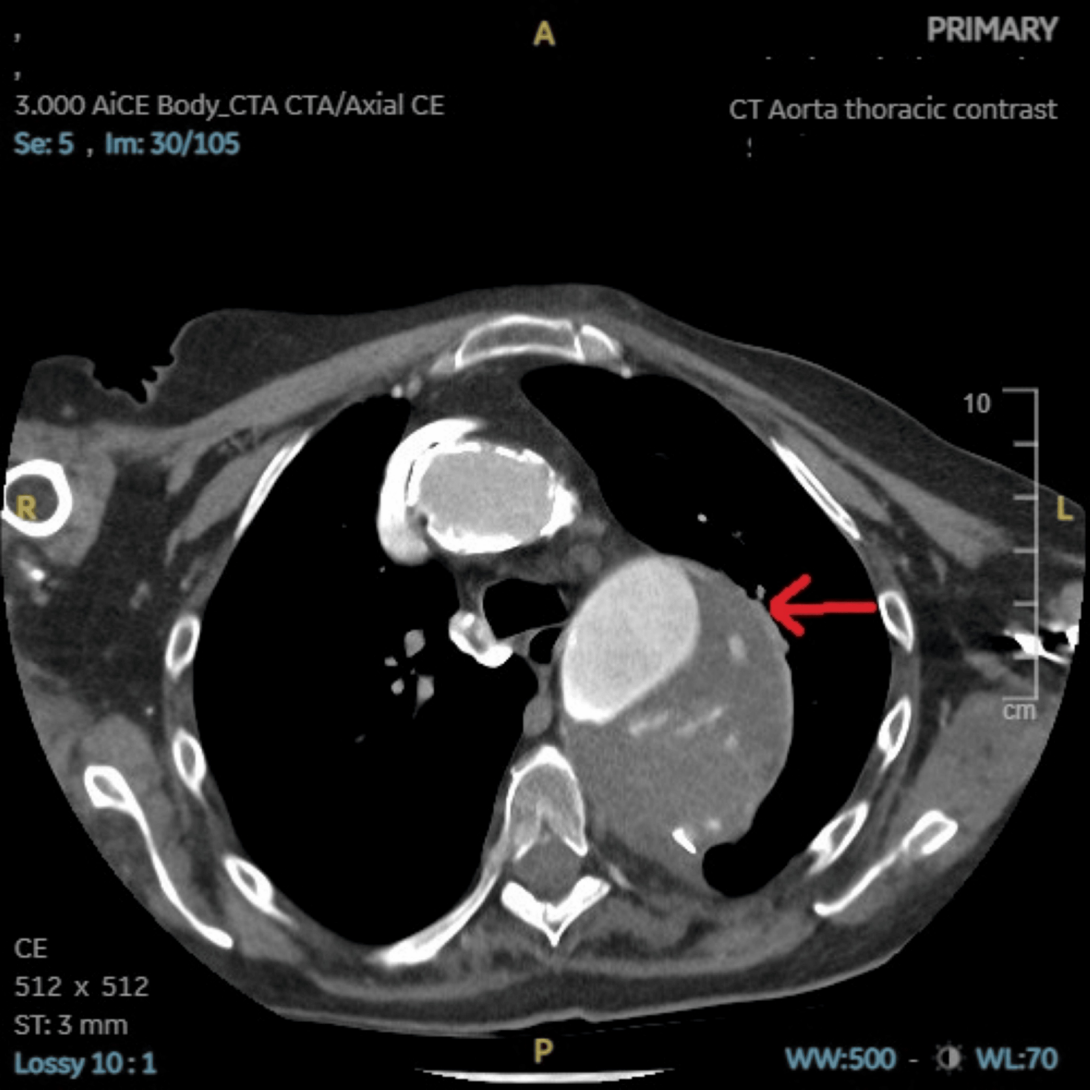
CT aorta with contrast axial view showing a large ruptured aneurysm as highlighted by the red arrow.

**Figure 5:**
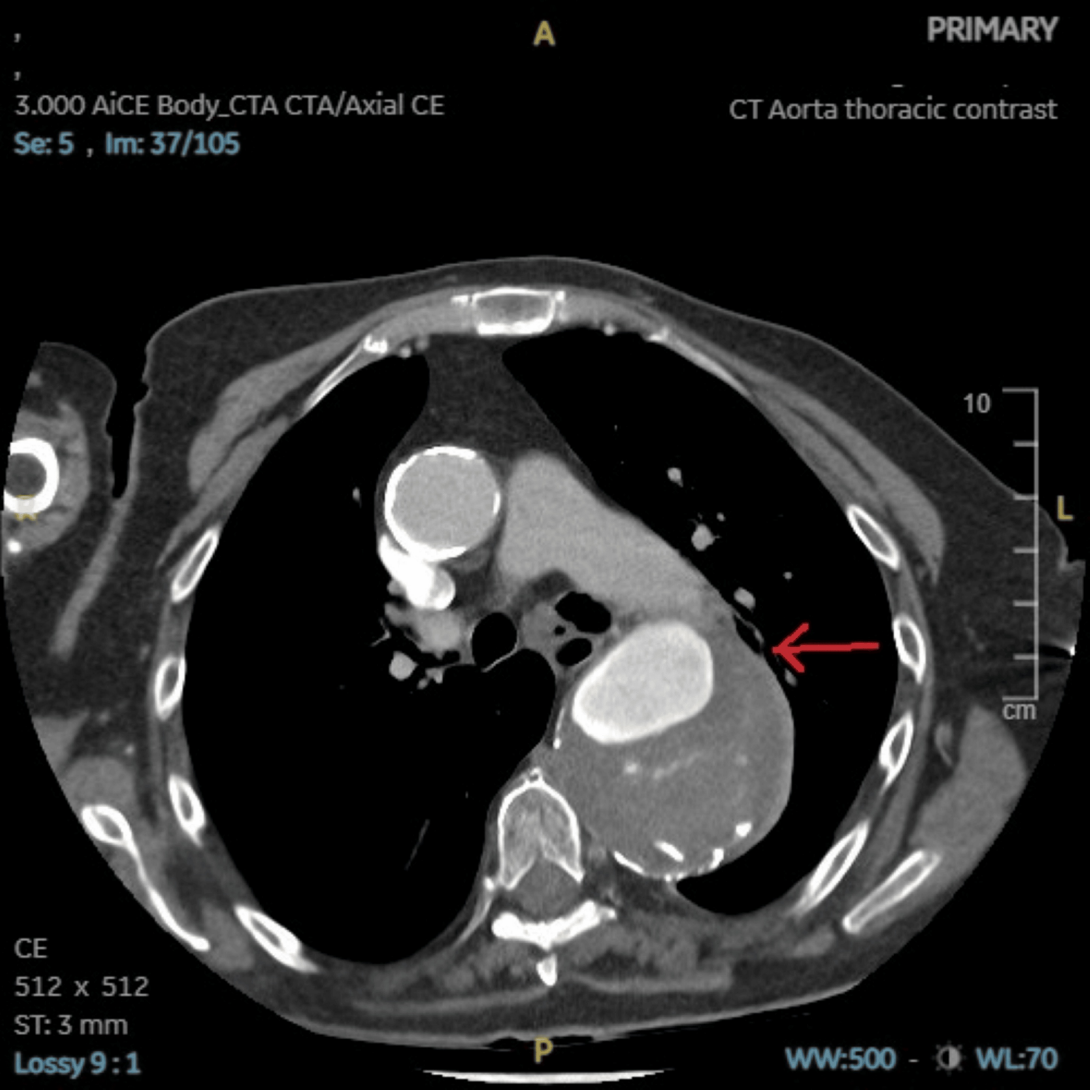
CT aorta with contrast axial view showing a large ruptured aneurysm as highlighted by the red arrow.

**Figure 6:**
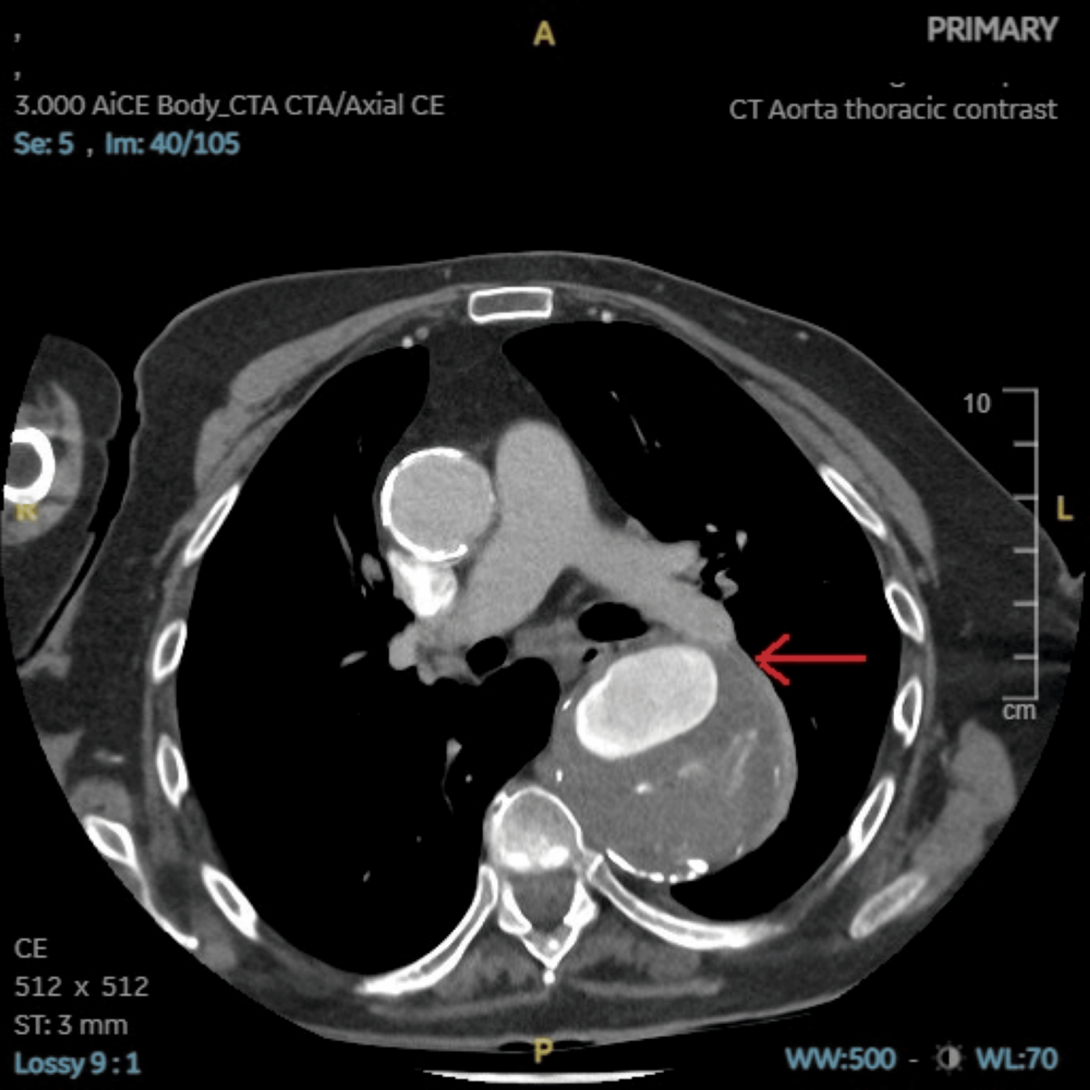
CT aorta with contrast axial view showing a large ruptured aneurysm as highlighted by the red arrow.

**Figure 7:**
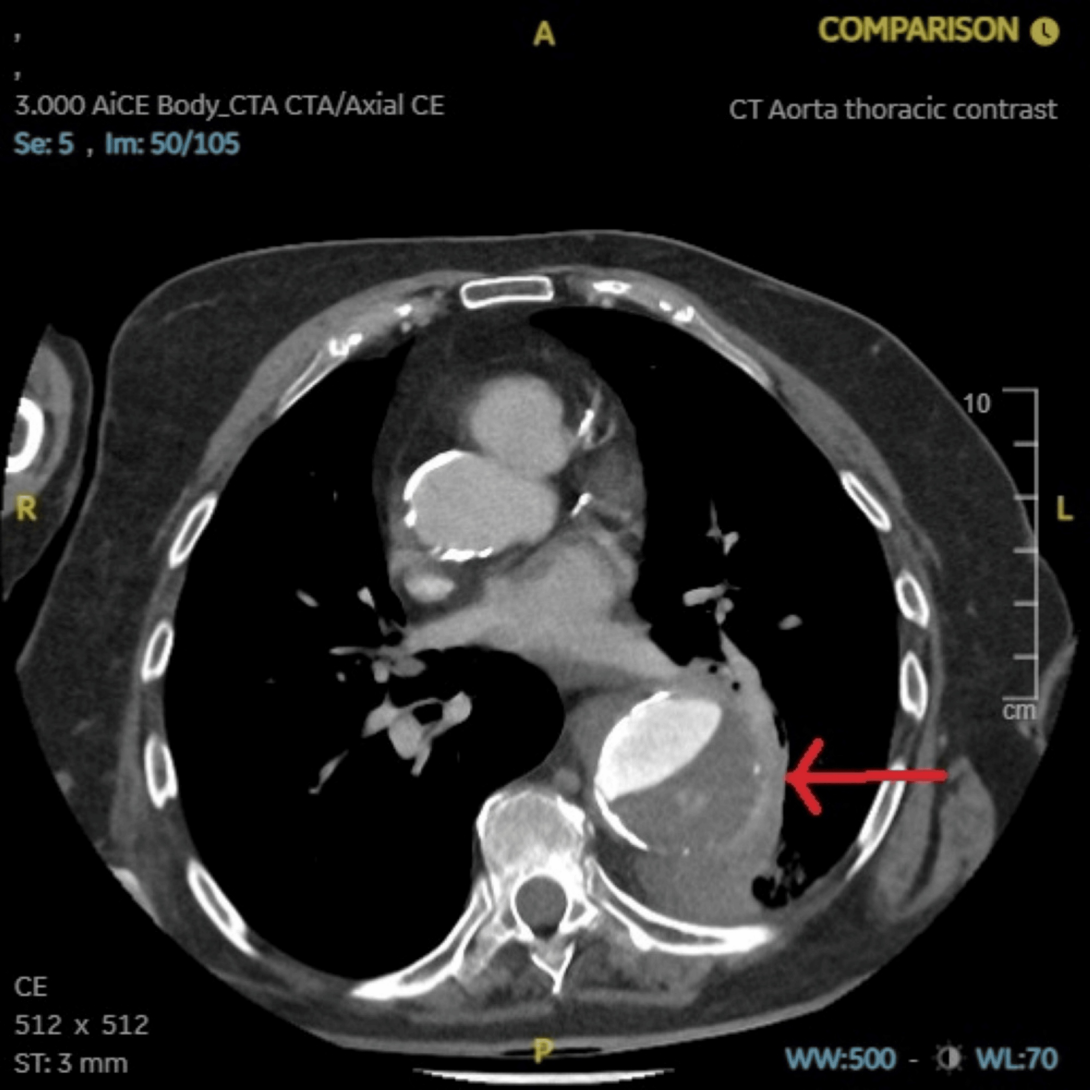
CT aorta with contrast axial view showing a 78 x 78 mm large ruptured aneurysm of the descending aorta as highlighted by the red arrow.

**Figure 8:**
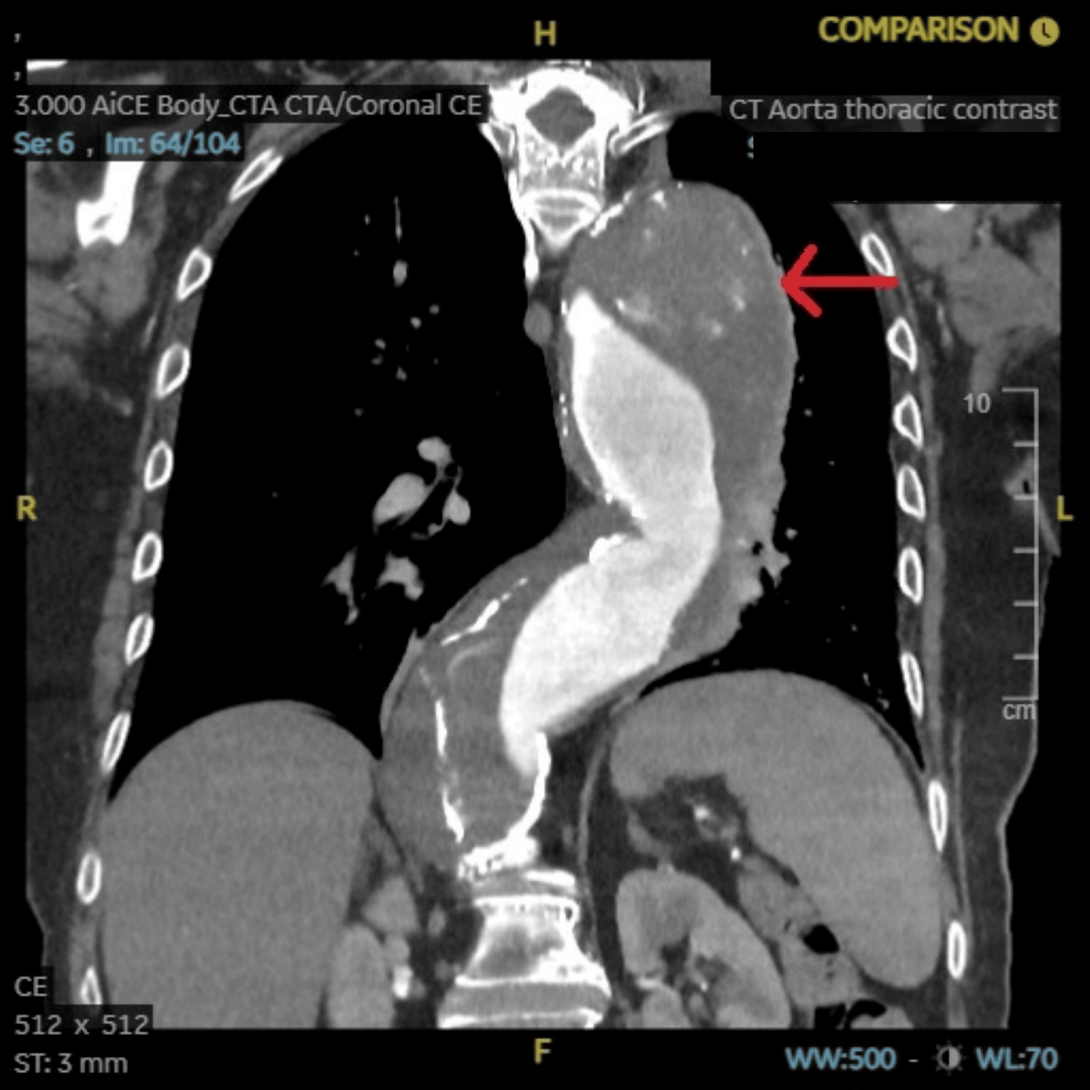
CT aortogram with contrast axial view showing a 78 x 78 mm large ruptured aneurysm of the descending aorta as highlighted by the red arrow.

Following physical examination, several investigations were requested, including a blood panel (Table 1), an electrocardiogram (ECG) (Table 2), a computed tomography of the head (CT head) (Table 2), a chest X-ray (Table 2, Figure 1), and a urine dip (Table 2). Her examination and an unremarkable CT head, ECG, and urine dip narrowed down the differential diagnosis of acute delirium, most likely due to a respiratory cause, including respiratory tract infection or aspiration pneumonia. Immediately after the CT head at around 11:45 (11:45 am), a chest X-ray was performed, which showed a massive TAA, which was alerted by the reporting radiologist. Following this, a CT aortogram with contrast (Table 2, Figures 2-8) was requested, which confirmed the diagnosis of ruptured TAA.

**Table 1:** Blood investigations that were taken at the time of presentation

Bloods	Results	Reference values
Haemoglobin	142	135-160 (g/L)
White blood cell	11.0	4.0-11.0 (*10^9/L)
Neutrophils	8.50	2.0-7.0 (*10^9/L)
Platelet	353	150-400 (*10^9/L)
C-reactive protein	62	<5 (mg/L)
Sodium	140	135-145 (mmol/L)
Potassium	3.5	3.5-5.5 (mmol/L)
Urea	3.5	2.0-7.0 (mmol/L)
Creatinine	110	55-120 (mmol/L)
Glomerular filtration rate	90	90-200 (mL/min)
Albumin	28	30-50 (g/L)
Adjusted calcium	2.66	2.1-2.6 (mmol/L)
pH	7.358	7.35-7.45
Partial pressure of CO2	7.32	4.5-6.0 (kPa)
Partial pressure of O2	1.62	10-14 (kPa)
Bicarbonate	30.9	22-28 (mmol/L)
Lactate	1.6	0.5-2.2 (mmol/L)
Base excess	3.8	-2 to +2 (mmol/L)
Creatine kinase	53	25-200 (U/L)
Glucose	5.9	3.0-6.0 (mmol/L)

**Table 2:** Other investigations in the form of radiological investigations and electrocardiography at the time of presentation.

Investigations	Report
Electrocardiography	Sinus rhythm with right bundle branch block
X-ray chest	Large thoracic aortic aneurysm in the region of the aortic arch, which was not evident on previous examination
CT head	No acute intracranial abnormality.
CT aortogram with contrast	78 x 78 mm large ruptured aneurysm of the descending aorta. Small volume fluid at the site of a large descending thoracic aneurysm has blood.

Subsequent to the preliminary investigations, the patient was referred to the vascular team and was relocated to the resuscitation bay for intensive monitoring. During this period, she was given intravenous labetalol to lower her systolic blood pressure to around 110 mm Hg. The vascular team initially considered surgical options, such as thoracic endovascular aortic repair; however, due to the patient's age and existing comorbidities, they chose conservative medical management for the ruptured aneurysm. During her five-day hospitalisation, she maintained a stable condition and was subsequently discharged with plans for outpatient follow-up care provided by community vascular nurses.

## Discussion

Aortic arch syndrome encompasses a collection of potentially fatal disorders affecting the aorta, such as aortic dissection, intramural hematoma without an intimal tear, penetrating atherosclerotic ulcer, and either impending or ruptured aortic aneurysm [5]. This discussion will concentrate on aortic aneurysm, encompassing both thoracic and abdominal aortic aneurysms.

Aortic aneurysms are primarily classified into two main types: TAA and abdominal aortic aneurysm (AAA), with the diaphragm serving as the defining boundary between the two. TAA can further be divided based on the anatomical location, which includes the ascending arch or descending sections of the aorta. The Crawford and Coselli classification system is widely recognised for categorising thoraco-abdominal aortic aneurysms as it takes into account both the extent of aneurysm and the surgical approach required for treatment (Table 3) [6]. In this classification, Type I refers to the descending thoracic aorta extending from the origin of the left subclavian artery to the suprarenal abdominal aorta, Type II pertains to the descending thoracic aorta from the subclavian artery to the aortoiliac bifurcation, Type III encompasses the distal thoracic aorta up to the aortoiliac bifurcation, and Type IV includes the abdominal aorta situated below the diaphragm (Table 3) [6]. Safi's team made an adjustment to this framework by incorporating Type V, which extends from the distal thoracic aorta, encompassing the celiac and superior mesenteric origins, but excluding the renal arteries (Table 3) [7].

**Table 3:** Crawford classification of thoraco-abdominal aneurysms Source: [6,7]

Types	Anatomical description
Type 1	Involvement of the entire descending thoracic aorta distal to the left subclavian artery, involving only the upper abdominal aorta superior to the renal arteries
Type 2	Involvement of the entire descending thoracic aorta distal to the left subclavian artery and most, if not the entire abdominal aorta
Type 3	Involvement of the descending aorta from the sixth intercostal space to include the renal arteries and most if not the entire abdominal aorta
Type 4	Involvement of the entire abdominal aorta with sparing of the thoracic aorta
Type 5	Involvement of the descending aorta from the sixth intercostal space to just superior to the renal arteries

The histopathological characteristics of thoraco-abdominal aortic aneurysm involve degradation or abnormality of collagen or elastin within the medial layer of the aortic wall. This degradation leads to subsequent dilatation, which is influenced by hemodynamic forces acting on the arterial wall, as well as intrinsic changes in the composition of the arterial wall itself [8]. Abdominal aortic aneurysms occur much more frequently than TAAs. The incidence of abdominal aortic aneurysm increases sharply after the age of 55 years in men and 70 years in women [9]. The prevalence of abdominal aortic aneurysms is approximately 5% in men aged 65 years and older who have undergone ultrasound screening [9]. By contrast, TAA has an incidence of 10 cases per 100000 patient years and a prevalence ranging from 0.16 to 0.34% [10].

Following the confirmation of an aortic aneurysm, the next course of action is to manage the patient based on the size and location of the aneurysm. Established guidelines exist for the surgical repair of TAAs (Table 4) [11]. General recommendations for aortic aneurysm management can also be applied (Table 5) [12].

**Table 4:** Indications of surgical repair of thoracic aortic aneurysm Source: [11]

Indication	Description
1	Rupture
2	Acute dissection resulting in malperfusion or other life-altering complications
3	Symptomatic states: Either pain consistent with rupture and unexplained by other causes OR compression of adjacent organs
4	Documented enlargement ≥1 cm/year or substantial growth approaching absolute size criteria
5	Absolute size >6.5 cm or >6.0 cm in patients with connective tissue disorders

**Table 5:** General recommendations for aortic aneurysm management Source: [12]

Recommendations	
1	For very small aneurysms, 3.0–3.9 cm, the risk of rupture is negligible. Therefore, these aneurysms do not require surgical intervention and should be kept under ultrasound surveillance at regular intervals.
2	For aneurysms between 4 and 5.5 cm in diameter, rapid growth (>1 cm/year) and the development of symptoms referable to the aneurysm warrant intervention.
3	Aneurysm repair should be considered at a maximum aneurysm diameter of 5.2 cm in females and 5.5 cm in males.

The medical approach to managing aortic aneurysms focuses on alleviating stress on the aorta and involves the use of various medications, including beta-blockers, angiotensin-receptor blockers, aspirin, and statins. In addition to pharmacological treatment, lifestyle changes are essential; these include smoking cessation, maintaining a healthy weight, and effectively managing conditions such as diabetes, chronic obstructive pulmonary disease, and hypertension [2,12,13]. If medical management is chosen, the patients should be regularly followed up on. However, if the situation warrants, surgical intervention may be necessary, depending on the aneurysm‘s location and extent.

In some instances with unusual presentations, the initial diagnosis considered was significantly different from the eventual diagnosis of a dissected TAA. One such atypical case involved a patient who was initially diagnosed with heart failure, exhibiting symptoms of shortness of breath and bilateral leg swelling, which was later confirmed by a trans-oesophageal echocardiogram to be caused by a TAA [14]. Another example involved a patient who was discharged after receiving symptomatic treatment for abdominal pain, vomiting, and diarrhea, only to return with similar symptoms along with scrotal swelling and a collapse that was presumed to be due to a large pericardial effusion, as indicated by a bedside echocardiogram. He was subsequently taken to the operating room for drainage, only to confirm the presence of a dissected TAA, resulting in a tragic outcome of death [15]. These cases highlight the importance of recognising how atypical presentations can resemble other medical conditions, which may lead to delays in appropriate management.

This study had limitations, including the unavailability of a trans-oesophageal echocardiogram.

## Conclusions

AAS, particularly a ruptured aortic aneurysm, ranks among the top causes of mortality linked to cardiovascular diseases. Its often asymptomatic or atypical nature underscores the necessity for healthcare professionals to maintain a high level of vigilance when assessing patients who may be at risk. As emphasised in this case report, it is crucial to consider a broad range of differential diagnoses, including AAS, in patients with dementia and unusual symptoms.

Ideally, enhancing clinical awareness regarding aortic aneurysms, coupled with the continuous advances in imaging techniques and management strategies, is essential for mitigating the associated morbidity and mortality associated with this condition. Additionally, implementing a collaborative approach that involved a multidisciplinary team, including emergency physicians, vascular surgeons, cardiothoracic surgeons, anaesthetists, and intensivists, should be prioritised to ensure optimal patient care from the outset.
